# Risk Factor Analysis and Predictive Nomogram for Heart Valve Calcification in Rheumatoid Arthritis

**DOI:** 10.31083/RCM38668

**Published:** 2025-09-28

**Authors:** Yiwei Lu, Xu Zhao, Xinyi He, Menglan Li, Qingqing Xie, Shiquan Shuai

**Affiliations:** ^1^Department of Rheumatology and Immunology, Nanchong Central Hospital (Nanchong Clinical Medical Research Center)—The Second Clinical Medical College of North Sichuan Medical College, 637000 Nanchong, Sichuan, China; ^2^Key Laboratory of Inflammation and Immunity Nanchong, Nanchong Central Hospital (Nanchong Clinical Medical Research Center)—The Second Clinical Medical College of North Sichuan Medical College, 637000 Nanchong, Sichuan, China

**Keywords:** rheumatoid arthritis, calcification of heart valves, prediction model

## Abstract

**Background:**

To develop a predictive model for cardiac valve calcification (CVC) in rheumatoid arthritis (RA) patients using a novel nomogram approach.

**Methods:**

We analyzed data from patients diagnosed with RA at the Department of Rheumatology and Immunology, Nanchong Central Hospital, between January 1, 2020, and October 31, 2023. Data were gathered on patient demographics, disease characteristics, laboratory tests, and imaging findings. Patients were randomly divided into a training set (n = 210) and a validation set (n = 140), in a ratio of 6:4, respectively. Least absolute shrinkage and selection operator (LASSO) regression was employed to identify risk predictors. Meanwhile, both single-factor and multi-factor logistic regression analyses were conducted to ascertain the risk factors associated with cardiac valve calcification. A predictive model was constructed using R software and validated through Bootstrap techniques. The performance of the model was evaluated using the area under the receiver operating characteristic (ROC) curve (AUC), calibration curves, and decision curve analysis (DCA).

**Results:**

A total of 350 RA patients were included in the study, of whom 67 (19.1%) were diagnosed with CVC. Multivariate analysis identified several significant risk factors for CVC, including hypertension (odds ratio (OR) = 15.496, 95% confidence interval (CI): 4.373–54.916; *p* < 0.01), age (OR = 1.118, 95% CI: 1.003–1.246; *p* = 0.043), disease duration (OR = 1.238, 95% CI: 1.073–1.427; *p* = 0.003), and elevated erythrocyte sedimentation rate (ESR) (OR = 1.026, 95% CI: 1.006–1.047; *p* = 0.012). The predictive model demonstrated excellent discriminatory performance, with an AUC of 0.9474 (95% CI: 0.9044–0.9903) in the training set. The model also showed strong internal validity (C-index = 0.947) and maintained robust performance in external validation (AUC = 0.9390; 95% CI: 0.8880–0.9893). Calibration analysis further confirmed the predictive accuracy and reliability of the model.

**Conclusion:**

The developed model can effectively identify RA patients at high risk for CVC. This tool provides a scientific basis for clinical decision-making and has significant potential for enhancing patient management and outcomes.

## 1. Introduction

Rheumatoid arthritis (RA) is a chronic autoimmune disease primarily characterized by symmetrical polyarthritis. In addition to joint involvement, RA often presents with systemic manifestations, including cardiovascular complications [1, 2]. Among these, cardiac valve calcification (CVC) has attracted increasing attention due to its significant impact on morbidity and mortality. CVC refers to the progressive deposition of lipids and calcium on the heart valves, leading to thickening, stiffening, and impaired valve function [3]. RA patients are at a substantially higher risk of developing CVC than the general population, with an approximately fourfold increase in prevalence [4, 5, 6, 7].

Although the precise mechanisms underlying CVC in RA remain unclear, chronic inflammation, endothelial dysfunction, and autoimmune-mediated vascular damage have been implicated [8, 9, 10]. Notably, rheumatoid nodules—granulomatous lesions typically associated with severe seropositive RA—may occasionally develop on heart valve cusps or annuli. These nodules are believed to promote localized inflammatory injury, fibrosis, and eventual calcification [11, 12]. In addition, clinical factors such as advanced age, prolonged disease duration, hypertension, and elevated C-reactive protein (CRP) or erythrocyte sedimentation rate (ESR) levels have also been linked to an increased risk of CVC [10].

However, in routine clinical settings, identifying RA patients at high risk for CVC remains challenging due to the absence of simple, disease-specific predictive tools. Therefore, this study aimed to develop and validate a predictive model for CVC in RA patients using a nomogram approach based on routinely available clinical and laboratory parameters. The proposed model is expected to assist clinicians in individualized cardiovascular risk assessment and to guide timely preventive interventions, ultimately improving the prognosis and quality of life in this patient population.

## 2. Methods and Materials

### 2.1 Study Population

Patients who were diagnosed with rheumatoid arthritis at the Department of Rheumatology and Immunology of Nanchong Central Hospital from January 1, 2020, to October 31, 2023, were enrolled as participants for this study.

Inclusion Criteria: (1) A confirmed diagnosis of rheumatoid arthritis, adhering to the 2010 American College of Rheumatology/European League Against Rheumatic Diseases criteria [13]. (2) Availability of complete case data and demonstrated good cooperation during hospitalization. (3) A minimum disease duration of one year. (4) The study included patients aged 18 to 88 years. (5) Patients with complete case data and stable follow-up records during hospitalization were included.

We excluded conditions that could independently influence heart valve morphology or inflammation markers, to isolate RA-related effects. Exclusion Criteria: (1) A history of congenital heart disease, heart valve disease, rheumatic heart disease, or infective endocarditis. (2) Cases of liver or renal failure. (3) Occurrences of tumors, cardiovascular diseases, severe trauma, or acute infections within the last week.

This retrospective analysis was approved by the Ethics Committee of Nanchong Central Hospital [2024 Review (040)]. All procedures were conducted in accordance with the Declaration of Helsinki.

### 2.2 Methodologies

#### 2.2.1 Data Collection

Collected data included demographic details (gender, age, duration of disease, history of hypertension) and clinical parameters. Venous blood was drawn on the day of admission for comprehensive laboratory tests: CRP, potassium, phosphorus, sodium, calcium, white blood cells (WBC), platelets (Plt), ESR, cyclic citrulline peptide antibody (CCP), rheumatoid factor (RF), complements C3, C1q, C4, immunoglobulin G (IgG), high-density lipoprotein (HDL), low-density lipoprotein (LDL), lipoprotein a, lipoprotein b, total cholesterol, triglycerides (TG), uric acid, hematocrit, hemocreatinine, cystatin C, albumin, pre-albumin and glucose. These markers were collected to explore possible systemic immune activity related to vascular and valvular pathology in RA, although their association with CVC remains uncertain. These samples were analyzed using a G9206 automatic biochemistry analyzer (Myriad Biomedical Co., Ltd., Shenzhen, China) and a Cobas E601 electrochemiluminescence immunoassay analyzer (Roche Diagnostics, Penzberg, Germany). Additionally, echocardiography and disease duration were recorded. Disease duration refers to the time since confirmed RA diagnosis. To ensure adequate representation of CVC cases and to maintain robust model validation despite the modest overall sample size, the data were randomly divided into a training set (60%) and a validation set (40%) using R software (R 4.3.1, Lucent Technologies, https://www.r-project.org/). The training set was utilized to construct the nomogram model, and the validation set was used to assess the model’s predictive efficacy.

#### 2.2.2 Criteria for Diagnosing Heart Valve Calcification

Diagnosis of heart valve calcification was performed using an EPIQ7 echocardiographic machine (Philips, Amsterdam, the Netherlands) by two experienced sonographers, each with over five years of cardiovascular imaging experience. Standard transthoracic echocardiographic views were employed, primarily including the parasternal long-axis view and the apical four-chamber view. Calcification was defined as the presence of one or more strong echogenic areas measuring >1 mm in diameter located on the aortic valve, mitral valve, or mitral annulus, persisting throughout the cardiac cycle and lacking acoustic shadowing [14]. The grayscale intensity threshold was used to identify dense echogenic foci indicative of calcific deposits, in line with established echocardiographic criteria.

#### 2.2.3 Statistical Methods

Statistical evaluation was performed using SPSS version 23.0 (IBM, Armonk, NY, USA) and R version 4.3.1 (Murray Hill, NJ, USA). For continuous variables that did not conform to a normal distribution, the normality was assessed using the Shapiro–Wilk test. In cases where the Shapiro–Wilk test indicated a non-normal distribution, we employed the Mann–Whitney U test to compare differences between two groups. The results of these variables were reported as median values with interquartile ranges (Q1, Q3). Categorical data were processed using the chi-square test and are shown as n (%). Least absolute shrinkage and selection operator (LASSO) regression analysis was employed to identify risk factors, while logistic regression analysis was utilized for verification. Lambda.min refers to the value of the penalty term λ that gives the minimum cross-validated error, ensuring optimal variable selection. Additionally, R language software was used to create a nomogram based on the results of the multifactor logistic regression analysis. The effectiveness, clinical utility, and calibration of the column-line graph model were scrutinized through the receiver operating characteristic (ROC) curve, decision curve analysis (DCA), and calibration curves, respectively. Statistical significance was established at *p* < 0.05.

## 3. Results

### 3.1 General Information Analysis

**Fig. 1.**
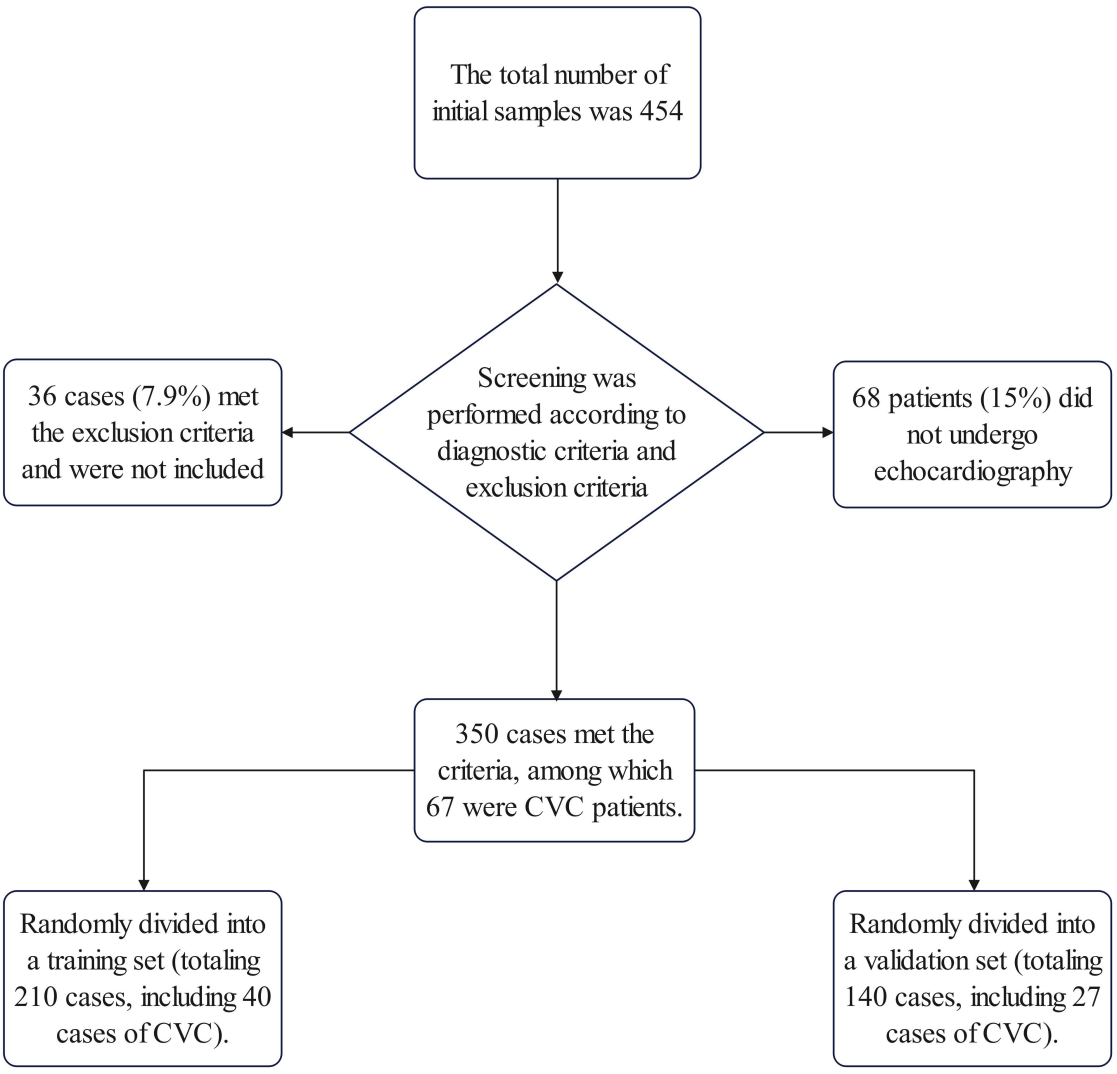
**Workflow of sample collection in this study**. CVC, cardiac valve calcification.

After screening according to the exclusion criteria, 350 patients were finally included. This cohort comprised 67 individuals (19.1%) with CVC and 283 (80.9%) without. Sixty-eight patients were excluded from the CVC analysis due to the absence of cardiac ultrasonography data, and an additional 36 were omitted based on exclusion criteria related to existing cardiac conditions and other factors (Fig. 1). The demographic breakdown showed 243 females (69%) and 107 males (31%), with a mean age of 68 (57, 73) years and an average disease duration of 10 (7, 13) years (Table 1). Within the CVC subgroup, there were 46 females (69%) and 21 males (31%), with 60 patients (89.6%) had hypertension.

**Table 1.** **Basic demographic information of RA patients**.

Variables		Total (n = 350)	RA patients without cardiac valve calcification CVC (n = 283)	RA patients with CVC (n = 67)	*p*
Hypertension, n (%)					<0.001
	Yes	88 (25)	28 (10)	60 (90)	
	No	262 (75)	255 (90)	7 (10)	
Sex, n (%)					0.879
	Male	107 (31)	86 (30)	21 (31)	
	Female	243 (69)	197 (70)	46 (69)	
Duration of disease, median (Q1, Q3)		10 (7, 13)	9 (6, 11)	15 (12.5, 18)	<0.001
Age, median (Q1, Q3)		68 (57, 73)	62 (55.5, 71)	75 (72, 78.5)	<0.001
K, median (Q1, Q3)		3.77 (3.47, 4.05)	3.77 (3.46, 4.06)	3.77 (3.5, 4.04)	0.862
P, median (Q1, Q3)		1.03 (0.91, 1.19)	1.03 (0.9, 1.19)	1.03 (0.92, 1.19)	0.832
Na, median (Q1, Q3)		140.6 (138.7, 142.4)	140.7 (138.8, 142.6)	139.8 (137.6, 141.9)	0.039
Ca, median (Q1, Q3)		2.15 (2.07, 2.23)	2.15 (2.07, 2.23)	2.17 (2.08, 2.22)	0.579
Plt, median (Q1, Q3)		252 (197, 333)	252 (197, 332)	252 (194, 336)	0.890
WBC, median (Q1, Q3)		7.03 (5.62, 9.32)	7.05 (5.69, 9.16)	6.94 (5.31, 9.37)	0.539
ESR, median (Q1, Q3)		68.5 (41, 99.8)	68.5 (39.5, 90.5)	92 (68.5, 112)	<0.001
CCP, median (Q1, Q3)		251.15 (251.15, 251.15)	251.15 (251.15, 251.15)	251.15 (251.15, 265.60)	0.472
RF, median (Q1, Q3)		260 (120.25, 473.25)	260 (111, 409)	260 (221, 619)	0.069
C3, median (Q1, Q3)		981.5 (873, 1070)	981.5(865, 1080)	981.5 (892, 1020)	0.505
C1q, median (Q1, Q3)		180 (161, 201)	180 (161, 201)	180 (160.5, 203.5)	0.869
C4, median (Q1, Q3)		219.5 (185, 255.8)	219.5 (185.5, 265.5)	219.5 (181, 230.5)	0.250
Lipoprotein B, median (Q1, Q3)		0.88 (0.77, 1.01)	0.88 (0.79, 1.03)	0.88 (0.71, 0.94)	0.273
Lipoprotein A, median (Q1, Q3)		167.65 (122.95, 252.35)	167.65 (115, 234.15)	167.65 (167.65, 314.95)	0.016
IgG, median (Q1, Q3)		13.7 (11.8, 16.4)	13.7 (11.7, 16)	13.7 (12.6, 18.8)	0.115
Cholesterol, median (Q1, Q3)		4.3 (3.72, 4.94)	4.3 (3.87, 5)	4.01 (3.33, 4.52)	0.003
HDL, median (Q1, Q3)		1.22 (1.02, 1.49)	1.22 (1.03, 1.50)	1.22 (0.97, 1.42)	0.413
LDL, median (Q1, Q3)		2.41 (1.99, 2.89)	2.42(2.08, 2.92)	2.21 (1.81, 2.64)	0.006
TG, median (Q1, Q3)		1.15 (0.87, 1.67)	1.15 (0.89, 1.67)	1.15 (0.84, 1.67)	0.691
UA, median (Q1, Q3)		270 (220.1, 337)	269 (220.1, 325.3)	284.5 (219.6, 361.7)	0.187
Urea, median (Q1, Q3)		5.15 (4.01, 6.69)	5.12 (3.93, 6.45)	5.73 (4.53, 7.61)	0.021
Cystatin C, median (Q1, Q3)		1.24 (1.00, 1.65)	1.21 (0.97, 1.55)	1.48 (1.11, 1.82)	<0.001
Cr, median (Q1, Q3)		56.9 (47, 69)	56.2 (47, 67.5)	60 (47, 75.3)	0.144
Albumin, mean ± SD		38.51 ± 4.29	38.75 ± 4.21	37.49 ± 4.49	0.040
Prealbumin, median (Q1, Q3)		180 (135, 231)	186 (136, 235.5)	169 (134.5, 220.5)	0.183
CRP, median (Q1, Q3)		38.4 (11.0, 68.7)	38 (9.6, 64.9)	44.4 (15.4, 76.7)	0.119
Glu, median (Q1, Q3)		5.61 (4.86, 6.82)	5.50 (4.76, 6.79)	5.98 (5.42, 7.86)	0.001

CVC, cardiac valve calcification; RA, rheumatoid arthritis; ESR, erythrocyte sedimentation rate; CRP, C-reactive protein; RF, rheumatoid factor; CCP, cyclic citrullinated peptide antibody; WBC, white blood cells; Plt, platelets; IgG, immunoglobulin G; HDL, high-density lipoprotein; LDL, low-density lipoprotein; TG, triglycerides; UA, uric acid; Cr, creatinine; Na, sodium; K, potassium; Ca, calcium; P, phosphorus; Glu, glucose.

In addition to the identification of calcification, key echocardiographic parameters were analyzed to further characterize valvular and cardiac function in RA patients. Among patients with cardiac valve calcification, the average thickness of the aortic valve was 2.8 ± 0.4 mm, while that of the mitral valve leaflets was 3.1 ± 0.5 mm. The mean transvalvular pressure gradient across the aortic valve was 12.3 ± 4.7 mmHg, and across the mitral valve was 7.6 ± 3.2 mmHg. Diastolic function was also evaluated, with the average E/E^′^ ratio measured at the mitral annulus being 12.7 ± 3.8 in the CVC group, compared to 9.3 ± 2.6 in patients without CVC (*p* < 0.001), suggesting higher left ventricular filling pressures in the calcification group. No cases of moderate or severe valvular stenosis were observed, and left ventricular ejection fraction remained within normal range in both groups.

### 3.2 LASSO Regression Analysis and Cross-Validation of Risk Factors for CVC in RA Patients

**Fig. 2.**
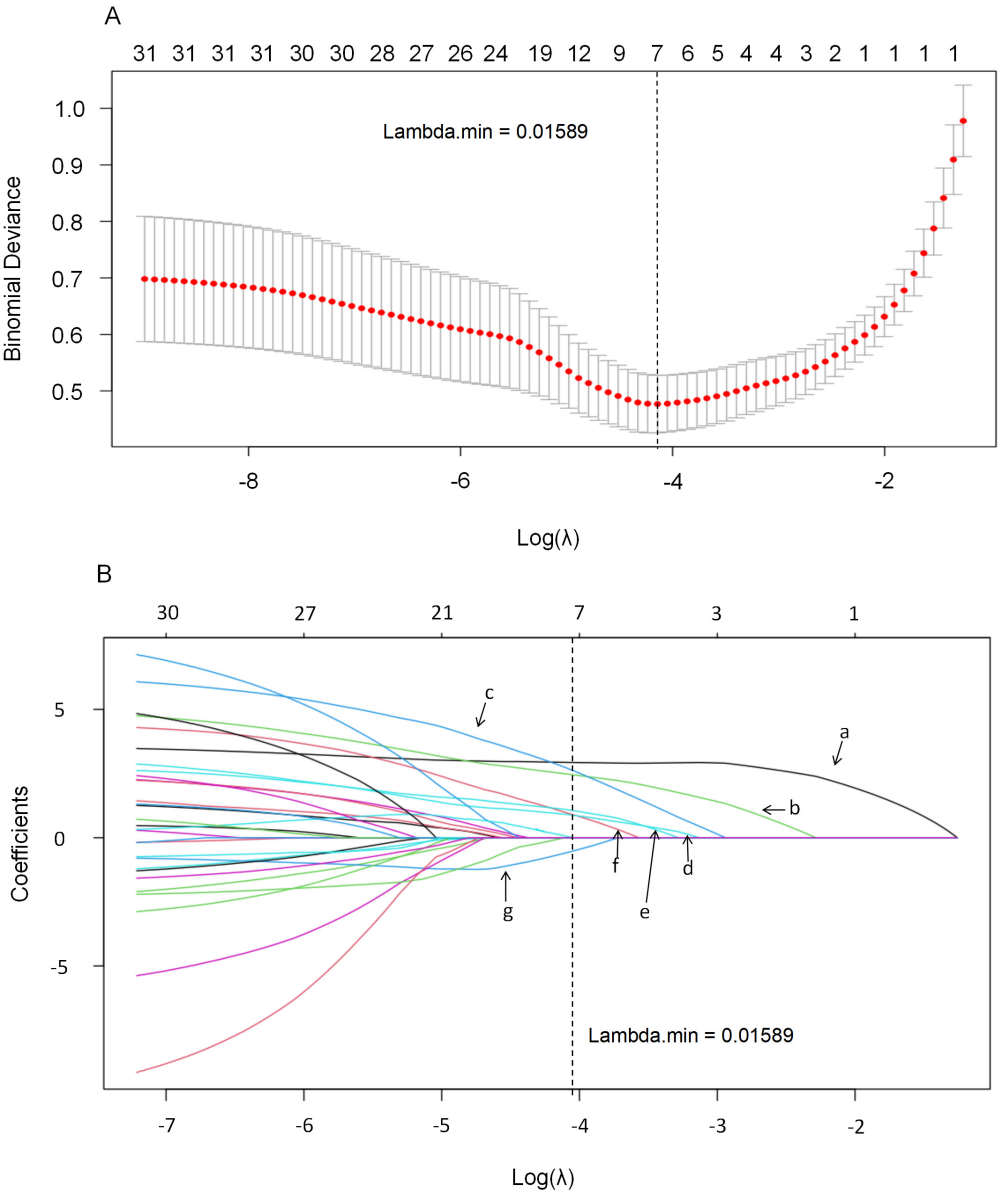
**This figure illustrates the application of the LASSO regression model for variable selection within the cohort**. The model employs a 10-fold cross-validation method (A) to enhance its reliability. Using Lambda.min (B) as the cutoff point, the analysis identifies seven predictors with non-zero coefficients: (a) Hypertension; (b) Duration of disease; (c) Age; (d) ESR; (e) Urea; (f) Cystatin C; and (g) Cr. Cr, creatinine. LASSO, least absolute shrinkage and selection operator.

LASSO regression was applied to select variables with non-zero coefficients that may be predictive of CVC. The Lambda.min value (0.01589) was determined through 10-fold cross-validation to minimize binomial deviance. Selected variables were subsequently entered into univariate and multivariate logistic regression analyses. The 31 variables mentioned were included in the LASSO regression analysis, with Lambda.min serving as the cutoff point for screening the independent variables. At a Lambda value of 0.01589, seven variables with non-zero coefficients were identified: age, disease duration, hypertension, ESR, urea, cystatin C, and creatinine. The LASSO regression system profile and cross-validation results are illustrated in Fig. 2.

### 3.3 Logistic Regression Analysis and Construction of Nomogram

**Fig. 3.**
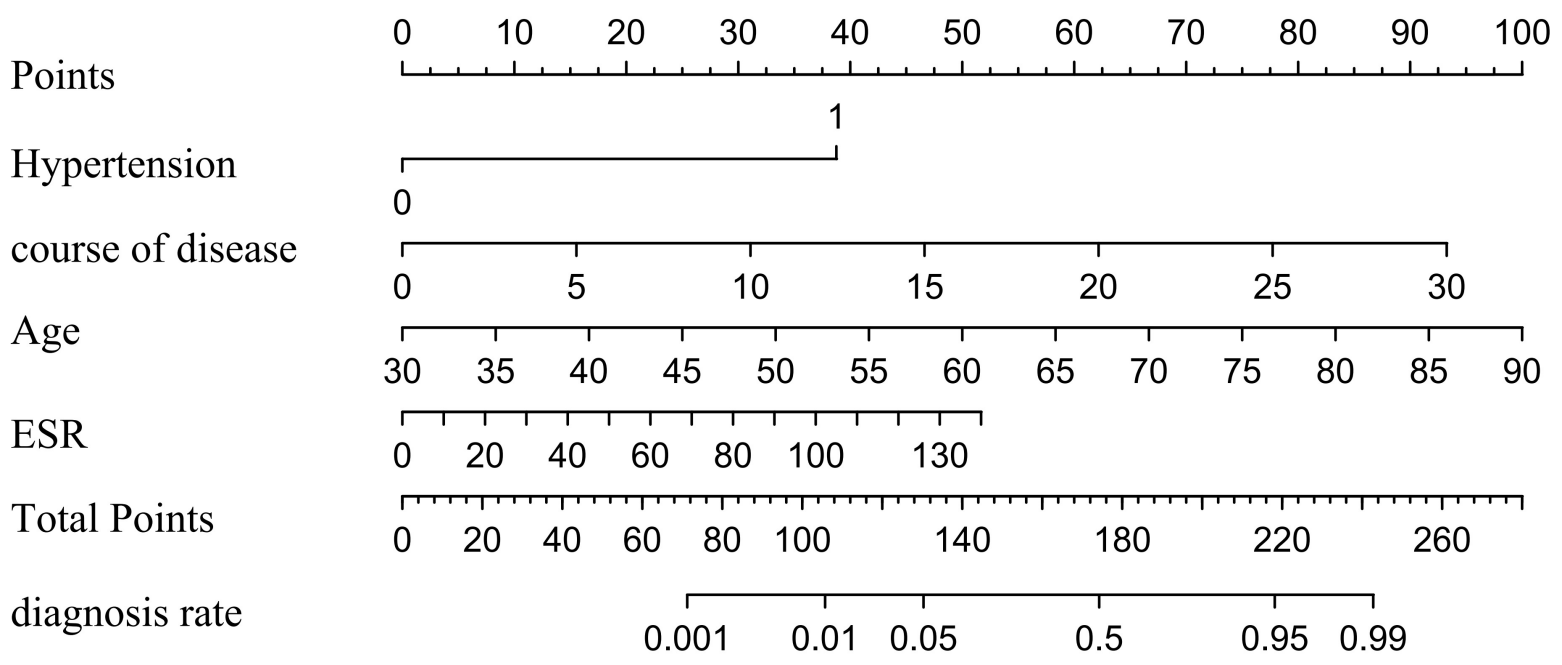
**Based on the results of multi-factor logistic analysis, predicting the probability of CVC in RA patients**. CVC, cardiac valve calcification; RA, rheumatoid arthritis; ESR, erythrocyte sedimentation rate.

The analysis began with a univariate logistic regression using data from the training set, with the presence of CVC as the outcome variable (coded as no = 0, yes = 1). Significant predictors included age, disease duration, hypertension, ESR, urea, cystatin C, and creatinine (all *p* < 0.05), as detailed in **Supplementary Table 1**. Of the seven variables selected, four remained statistically significant (*p* < 0.05) in the multivariate logistic regression model, while the others were excluded due to lack of significance. A subsequent multivariate logistic regression incorporated these variables (gender coded as male = 0, female = 1) and confirmed age, disease duration, hypertension, and ESR as independent predictors of CVC (Table 2). These findings were used to construct a nomogram predicting the probability of CVC in RA patients (Fig. 3).

**Table 2.** **Multivariate logistic regression analysis results**.

Predictor variable	B	SE	Wald value	*p*-value	OR value	95% CI
Disease duration	0.213	0.073	8.588	0.003	1.238	1.073–1.427
Age	0.111	0.055	4.080	0.043	1.118	1.003–1.246
Hypertension	2.741	0.646	18.024	<0.01	15.496	4.373–54.916
ESR	0.026	0.011	6.243	0.012	1.026	1.006–1.047
Cystatin C	0.451	0.504	0.802	0.37	1.570	0.585–4.213
Urea	0.107	0.141	0.576	0.448	1.113	0.844–1.468

OR, odds ratio; CI, confidence interval; ESR, erythrocyte sedimentation rate.

**Fig. 4.**
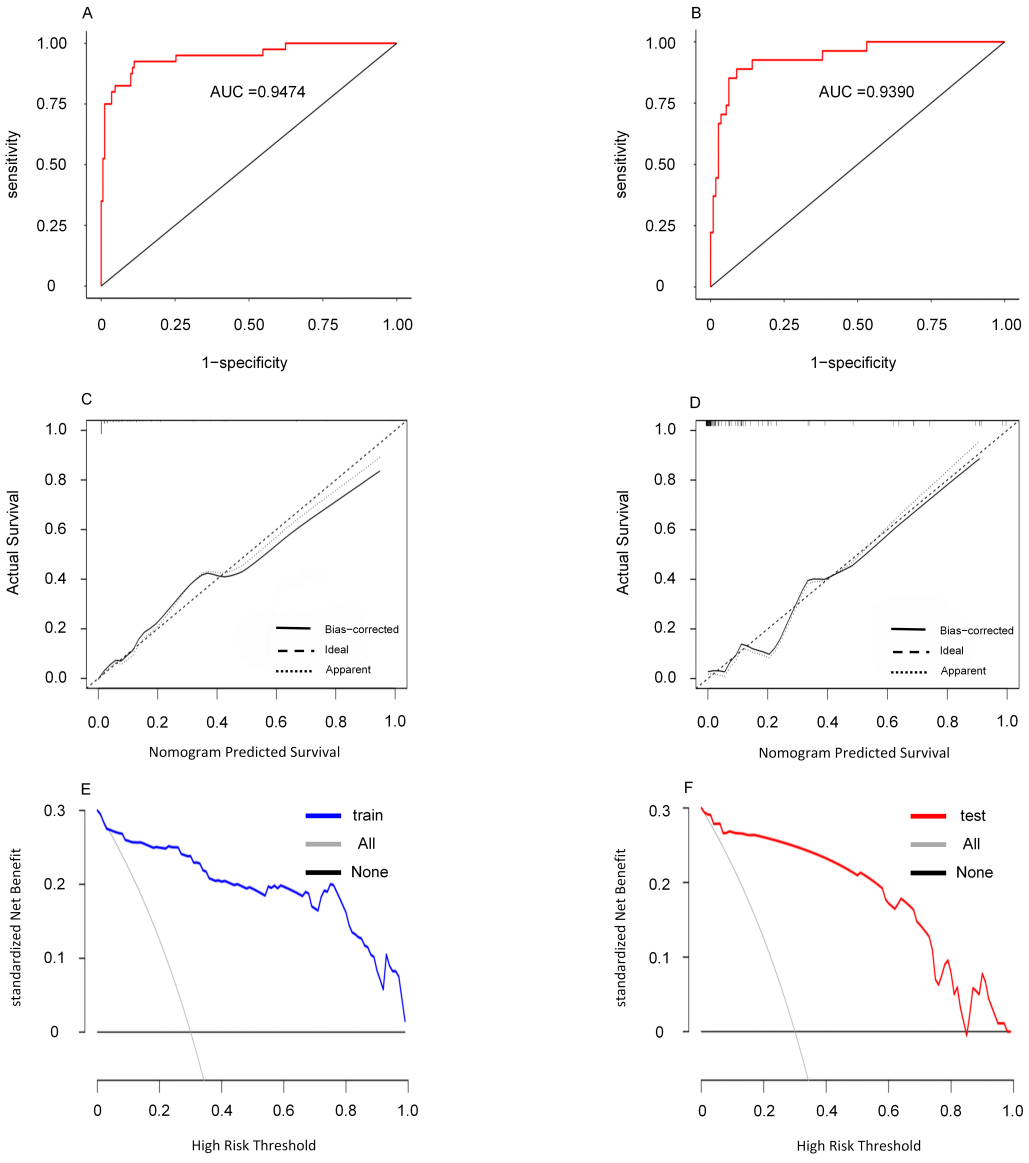
**This figure presents an evaluation of the nomogram, including the ROC curve (A), calibration curve (B), calibration curve (C), the ROC curve (D), decision curve (E) and decision curve (F) are shown for the validation centralized nomogram**. In the decision curve, the term ‘all’ denotes the scenario in which all patients develop CVC, while ‘None’ indicates the scenario where no patients develop CVC. CVC, cardiac valve calcification; ROC, receiver operating characteristic; AUC, area under the receiver operating characteristic curve.

The predictive performance of the model was validated with ROC curves, achieving an area under the receiver operating characteristic curve (AUC) of 0.9474 (95% CI: 0.9044–0.9903) for the training set and 0.9390 (95% CI: 0.8880–0.9893) for the validation set. Calibration curves displayed excellent agreement between observed occurrences and predictions, while DCA indicated substantial clinical utility and net benefit across a wide score range (Fig. 4).

## 4. Discussion

Despite notable advancements that have reduced overall mortality among RA patients, there remains a concerning increase in mortality associated with concomitant cardiovascular diseases (CVD). This trend underscores the critical need for early diagnosis and proactive intervention in the precision medicine era. In this study, we have identified age, disease duration, hypertension, and blood sedimentation rate as independent risk factors for CVC in RA patients, utilizing both LASSO regression model, univariate and multivariate logistic regression analyses. A nomogram model was further constructed to predict the risk of CVC in RA patients, aiming to identify patients at high risk of CVC as early as possible.

Savage *et al*. [15] found in early studies that as age increases, the risk of CVC also rises, with a positive correlation between prevalence and age. In the general population, some studies suggest that the formation of CVC may be influenced by several factors, including passive calcium deposition, lipid deposition, and changes in hemodynamics [16]. This study found that the age of the group with valve calcification was generally higher than that of the group without valve calcification. While this does not exclude the possibility that the observed differences may be attributed to the aforementioned factors, it also suggests that inflammatory stimuli may contribute to the formation of CVC. Furthermore, as previously mentioned, CVC serves as a risk marker for cardiovascular disease, potentially leading to earlier occurrences of cardiovascular events compared to the general population. Further research by Fulkerson *et al*. [17] indicates that CVC may lead to severe cardiovascular conditions such as valvular stenosis [18], endocarditis [19], atrial fibrillation, and atrioventricular block [15, 20], with a noted potential for triggering cerebrovascular events [21]. These findings highlight the importance of early and precise assessment and intervention for heart valve issues in RA patients. By doing so, we can possibly slow the disease’s progression and, in some instances, decrease the incidence of both cardiovascular and cerebrovascular complications. Therefore, implementing regular and thorough cardiovascular assessments for RA patients—especially those who present with the identified risk factors—can be crucial in mitigating the risk of severe complications. This strategic approach not only helps in managing the immediate health concerns but also contributes to the long-term well-being and quality of life of the patients, thereby aligning with the goals of modern precision medicine.

Historical histopathologic studies have revealed that valvular calcification can develop in the context of systemic inflammation, where prolonged inflammatory states contribute to lipid deposition, macrophage and T-cell infiltration, and ultimately, disruption of the basement membrane—factors that collectively initiate the production of CVC [22]. It has been noted that an extended duration of disease significantly elevates the risk for CVC among RA patients. This observation is supported by findings from Yiu *et al*. [10], which indicated a marked increase in CVC prevalence when disease duration exceeded ten years. Consequently, RA patients with CVC exhibit a considerably higher likelihood of experiencing future cardiovascular events compared to the general population, potentially leading to severely diminished prognosis and quality of life [17, 18, 19, 20, 21]. Thus, early screening for cardiovascular complications and thorough cardiac function assessments are critical for RA patients who have lived with the disease for a decade or more. These measures are vital not only for managing current health concerns but also for implementing timely interventions to prevent severe cardiovascular diseases.

The precise mechanisms through which hypertension contributes to CVC remain somewhat elusive but are thought to involve sustained high blood flow against valve surfaces, increasing transvalvular pressure that over time may cause structural damage including fiber breakage and calcium salt deposition. This theory is corroborated by a comprehensive meta-analysis of cross-sectional and case-control studies involving over 6450 hypertensive patients, which suggested a potential link between high blood pressure and the development of CVC [11]. In our cohort, an overwhelming majority (89.6%) of patients diagnosed with CVC also had hypertension, echoing the broader data trends and suggesting that hypertension may exacerbate the progression of cardiovascular diseases in those already compromised by RA. Consequently, rigorous blood pressure monitoring and holistic management of both hypertension and CVC are imperative in the treatment protocol for RA patients to mitigate the risk of further cardiovascular complications.

Although hypertension is a recognized independent risk factor for cardiac valve calcification in the general population, its impact in RA patients may be compounded by chronic systemic inflammation and immune-mediated endothelial injury. In RA, prolonged exposure to elevated cytokine levels, oxidative stress, and vascular dysfunction may accelerate the deleterious effects of high blood pressure on valvular tissue. Our study revealed that 90% of RA patients with CVC had coexisting hypertension, supporting the hypothesis that inflammatory and hemodynamic factors may synergistically promote calcification in this unique clinical setting. Therefore, the contribution of hypertension in RA patients cannot be interpreted in isolation but should be considered within the broader context of autoimmune-mediated cardiovascular risk.

In this study, ESR has been identified as a potential risk factor for CVC in patients with RA. Elevated ESR levels are often observed in RA patients, likely reflecting ongoing disease activity and inflammation levels. Previous research by Ingelsson *et al*. [23] in a longitudinal cohort study suggested that high ESR is a significant predictor of heart failure, a finding further supported by Maradit-Kremers *et al*. [24], who reported that prolonged inflammatory stimuli in RA patients might lead to heart failure. These studies imply a possible link between elevated ESR and the development of CVC, although direct correlations are yet to be extensively documented. Our results open new avenues for research into the mechanisms underlying the relationship between ESR and CVC. Going forward, we aim to delve deeper into this association to devise more accurate diagnostic and preventative strategies for cardiovascular complications in RA patients. This study also highlights the importance of vigilant monitoring of ESR levels in clinical practice, which could be crucial for the timely detection and prevention of CVC.

Interventions aimed at delaying the progression of valve calcification in RA should address both inflammation and traditional cardiovascular risk factors. Biologic disease-modifying antirheumatic drugs (DMARDs), such as TNF-α inhibitors or IL-6 receptor antagonists, have shown efficacy in controlling systemic inflammation and may potentially reduce cardiovascular complications. In addition, statins may exert pleiotropic anti-inflammatory and anti-calcific effects, while strict blood pressure control using renin-angiotensin system inhibitors or calcium channel blockers may help to mitigate hemodynamic stress on the valves. Although these strategies require further prospective validation in RA-specific cohorts, they offer a practical foundation for preventing or slowing CVC development.

Numerous studies have investigated the potential link between CRP and CVC, yet this study did not establish a clear association between them. This outcome introduces both challenges and opportunities for further research, underscoring the need to delve deeper into the underlying causes and potential mechanisms. To enhance our understanding of this complex relationship, more comprehensive studies are required. Firstly, increasing the sample size would allow for a better representation of the general population and could help validate the findings more robustly. Secondly, employing advanced research techniques and tools, such as genomics and proteomics, could provide greater insights into the biochemical interactions at play. Additionally, conducting longitudinal studies to monitor CRP levels over time relative to CVC development could offer valuable data on how these factors correlate longitudinally. By expanding the scope of our research and employing these methods, we aim to uncover nuanced details about the relationship between CRP and CVC. This could potentially lead to innovative approaches for the prevention and treatment of CVC, thereby contributing to better cardiovascular health outcomes.

Importantly, the predictive model developed in this study incorporates both traditional cardiovascular risk factors and RA-specific indicators, such as ESR and disease duration, which reflect systemic inflammation and autoimmune burden. While hypertension and age are well-established risk factors for valve calcification, their inclusion alongside RA-specific markers adds novel clinical value. The use of a nomogram—a visual, user-friendly tool—allows for individualized risk estimation in clinical practice, facilitating early identification and intervention in high-risk RA patients. To our knowledge, this is the first study to construct such an integrative and quantitative model tailored to RA populations, addressing a critical gap in cardiovascular risk stratification for this group.

This research has its limitations that must be acknowledged. Primarily, being a single-center study with a relatively small sample size and data collected at a single point in time, there is a potential for bias which might affect the generalizability of the findings. Furthermore, while CRP is recognized as a marker for cardiovascular issues [12, 25, 26], our analysis did not find a definitive link between CRP levels and CVC. This could be due to several factors including the scale of the sample size, the particular characteristics of the study population, and the data collection and processing methodologies employed. Additional studies are necessary to explore this relationship further, considering other potential confounding variables such as lifestyle, genetic predispositions, and environmental influences which could impact the dynamics between CRP and CVC. Although an association between CRP and CVC is hypothesized, the complexity of this relationship suggests that a deeper understanding is required to draw conclusive links. Given the retrospective, single-center design of the study, selection bias may have influenced the findings. Patients with incomplete clinical records or those demonstrating poor compliance during hospitalization were excluded, potentially resulting in the underrepresentation of individuals with more severe disease or atypical presentations. This may limit the generalizability of the nomogram to broader RA populations. Future studies should aim to validate this model prospectively across multiple centers with more diverse cohorts, and explore the integration of imaging or biomarker-based indicators to enhance its predictive performance.

## 5. Conclusion

Upon analyzing the data and refining our predictive model, we identified that age, disease duration, hypertension, and ESR are key predictors of CVC in RA patients. These factors are vitally important for pinpointing patients at high risk for CVC. This model provides a potentially valuable tool to aid clinical risk assessment, though further prospective validation is warranted.

## Data Availability

All data generated or analysed during this study are included in this. Further enquiries can be directed to the corresponding author.

## Abbreviations

CVC, cardiac valve calcification; RA, rheumatoid arthritis; ROC, receiver operating characteristic; DCA, decision curve analysis; CRP, C-reactive protein; BNP, B-type natriuretic peptide; hsTnT, hypersensitive troponin; WBC, white blood cells; Plt, platelets; ESR, erythrocyte sedimentation rate; CCP, cyclic citrulline peptide; RF, rheumatoid factor; IgG, immunoglobulin G; HDL, high-density lipoprotein; LDL, low-density lipoprotein; TG, triglycerides.

## Supplementary Material

Supplementary material associated with this article can be found, in the online version, at https://doi.org/10.31083/RCM38668.

## References

[1] Isomäki H (1992). Long-term outcome of rheumatoid arthritis. *Scandinavian Journal of Rheumatology. Supplement*.

[2] Wolfe F (1996). The natural history of rheumatoid arthritis. *The Journal of Rheumatology. Supplement*.

[3] Bedeir K, Kaneko T, Aranki S (2019). Current and evolving strategies in the management of severe mitral annular calcification. *The Journal of Thoracic and Cardiovascular Surgery*.

[4] Murphy L (2022). Cardiovascular disease risk in rheumatoid arthritis. *British Journal of Nursing*.

[5] Solomon DH, Karlson EW, Rimm EB, Cannuscio CC, Mandl LA, Manson JE (2003). Cardiovascular morbidity and mortality in women diagnosed with rheumatoid arthritis. *Circulation*.

[6] Hak AE, Karlson EW, Feskanich D, Stampfer MJ, Costenbader KH (2009). Systemic lupus erythematosus and the risk of cardiovascular disease: results from the nurses’ health study. *Arthritis and Rheumatism*.

[7] Maradit-Kremers H, Nicola PJ, Crowson CS, Ballman KV, Gabriel SE (2005). Cardiovascular death in rheumatoid arthritis: a population-based study. *Arthritis and Rheumatism*.

[8] Cavalcanti LRP, Sá MPBO, Perazzo ÁM, Escorel Neto AC, Gomes RAF, Weymann A (2020). Mitral Annular Calcification: Association with Atherosclerosis and Clinical Implications. *Current Atherosclerosis Reports*.

[9] Corrao S, Messina S, Pistone G, Calvo L, Scaglione R, Licata G (2013). Heart involvement in rheumatoid arthritis: systematic review and meta-analysis. *International Journal of Cardiology*.

[10] Yiu KH, Wang S, Mok MY, Ooi GC, Khong PL, Lau CS (2011). Relationship between cardiac valvular and arterial calcification in patients with rheumatoid arthritis and systemic lupus erythematosus. *The Journal of Rheumatology*.

[11] García-Patos V (2007). Rheumatoid nodule. *Seminars in Cutaneous Medicine and Surgery*.

[12] Bonfiglio T, Atwater EC (1969). Heart disease in patients with seropositive rheumatoid arthritis; a controlled autopsy study and review. *Archives of Internal Medicine*.

[13] Aletaha D, Neogi T, Silman AJ, Funovits J, Felson DT, Bingham CO (2010). 2010 Rheumatoid arthritis classification criteria: an American College of Rheumatology/European League Against Rheumatism collaborative initiative. *Arthritis and Rheumatism*.

[14] Ellouali F, Berkchi F, Elhoussni S, Bayahia R, Benamar L, Abouqal R (2015). Evaluation of the effect of duration on dialysis on echocardiographic parameters: a preliminary study. *Saudi Journal of Kidney Diseases and Transplantation*.

[15] Savage DD, Garrison RJ, Castelli WP, McNamara PM, Anderson SJ, Kannel WB (1983). Prevalence of submitral (anular) calcium and its correlates in a general population-based sample (the Framingham Study). *The American Journal of Cardiology*.

[16] Katsi V, Magkas N, Antonopoulos A, Trantalis G, Toutouzas K, Tousoulis D (2021). Aortic valve: anatomy and structure and the role of vasculature in the degenerative process. *Acta Cardiologica*.

[17] Fulkerson PK, Beaver BM, Auseon JC, Graber HL (1979). Calcification of the mitral annulus: etiology, clinical associations, complications and therapy. *The American Journal of Medicine*.

[18] Movva R, Murthy K, Romero-Corral A, Seetha Rammohan HR, Fumo P, Pressman GS (2013). Calcification of the mitral valve and annulus: systematic evaluation of effects on valve anatomy and function. *Journal of the American Society of Echocardiography*.

[19] Nestico PF, Depace NL, Morganroth J, Kotler MN, Ross J (1984). Mitral annular calcification: clinical, pathophysiology, and echocardiographic review. *American Heart Journal*.

[20] Nair CK, Runco V, Everson GT, Boghairi A, Mooss AN, Mohiuddin SM (1980). Conduction defects and mitral annulus calcification. *British Heart Journal*.

[21] Dietl CA, Hawthorn CM, Raizada V (2016). Risk of Cerebral Embolization with Caseous Calcification of the Mitral Annulus: Review Article. *The Open Cardiovascular Medicine Journal*.

[22] Otto CM, Kuusisto J, Reichenbach DD, Gown AM, O’Brien KD (1994). Characterization of the early lesion of ’degenerative’ valvular aortic stenosis. Histological and immunohistochemical studies. *Circulation*.

[23] Ingelsson E, Arnlöv J, Sundström J, Lind L (2005). Inflammation, as measured by the erythrocyte sedimentation rate, is an independent predictor for the development of heart failure. *Journal of the American College of Cardiology*.

[24] Maradit-Kremers H, Nicola PJ, Crowson CS, Ballman KV, Jacobsen SJ, Roger VL (2007). Raised erythrocyte sedimentation rate signals heart failure in patients with rheumatoid arthritis. *Annals of the Rheumatic Diseases*.

[25] Ridker PM (2016). A Test in Context: High-Sensitivity C-Reactive Protein. *Journal of the American College of Cardiology*.

[26] Jeevanantham V, Singh N, Izuora K, D’Souza JP, Hsi DH (2007). Correlation of high sensitivity C-reactive protein and calcific aortic valve disease. *Mayo Clinic Proceedings*.

