# Supplementary material for: Risk Factor Analysis and Predictive Nomogram for Heart Valve Calcification in Rheumatoid Arthritis
Source: Rev Cardiovasc Med. 2025 Sep 28;26(9):38668. doi: 10.31083/RCM38668 (PMC12516738; doi:10.31083/RCM38668)
Supplement: Supplementary file 1 [file 2153-8174-26-9-38668-s1.docx]

**Supplementary Table 1.** Univariate logistic regression analysis results.

| Predictor variable | OR value | 95% CI | P-value |
| --- | --- | --- | --- |
| Total cholesterol | 0.949 | 0.678~1.328 | 0.761 |
| Lipoprotein B | 1.346 | 0.303~5.975 | 0.696 |
| Lipoprotein A | 1.000 | 0.999~1.002 | 0.733 |
| Glu | 1.075 | 0.961~1.204 | 0.207 |
| Sex (female) | 0.677 | 0.301~1.525 | 0.347 |
| Prealbumin | 0.999 | 0.994~1.004 | 0.714 |
| Uric acid | 1.001 | 0.997~1.004 | 0.676 |
| Urea | 1.236 | 1.064~1.437 | 0.006 |
| Age | 1.233 | 1.146 to 1.325 | <0.001 |
| Creatinine（Cr） | 1.011 | 1.001~1.021 | 0.038 |
| Cystatin C | 1.473 | 1.054~2.060 | 0.023 |
| Hypertension | 63.000 | 21.770~182.315 | <0.001 |
| Course of disease | 1.358 | 1.223~1.509 | <0.001 |
| Albumin | 0.949 | 0.870~1.035 | 0.24 |
| WBC | 0.939 | 0.823~1.059 | 0.304 |
| TG | 1.326 | 0.846~2.079 | 0.219 |
| RF | 1.000 | 1.000~1.001 | 0.357 |
| Plt | 1.001 | 0.998~1.005 | 0.407 |
| P | 3.268 | 0.785~13.607 | 0.104 |
| LDL | 0.845 | 0.526~1.356 | 0.485 |
| K | 1.191 | 0.566~2.505 | 0.645 |
| IgG | 1.087 | 1.016~1.163 | 0.076 |
| HDL | 1.131 | 0.435~2.942 | 0.800 |
| ESR | 1.019 | 1.007~1.030 | <0.001 |
| CRP | 1.000 | 0.993~1.007 | 0.961 |
| CCP | 1.001 | 0.998~1.005 | 0.477 |
| Ca | 1.362 | 0.087~21.404 | 0.826 |
| C4 | 1.001 | 0.996~1.005 | 0.757 |
| C3 | 1.000 | 0.998~1.002 | 0.780 |
| C1q | 1.003 | 0.994~1.011 | 0.517 |
| Na | 0.946 | 0.861~1.040 | 0.253 |
